# An Attention-Residual Hybrid CNN for CT-Based Multiclass Classification of Alcohol-Related Liver Disease: Differential Diagnosis Against HBV-Related Cirrhosis

**DOI:** 10.3390/diagnostics16182964

**Published:** 2026-09-14

**Authors:** Ertugrul Karabulut, Mucahit Karaduman, Muhammed Yildirim, Sami Akbulut

**Affiliations:** 1 Department of Surgery and Liver Transplantation, Faculty of Medicicne, Inonu University, 44280 Malatya, Türkiye; 2 Department of Software Engineering, Malatya Turgut Ozal University, 44210 Malatya, Türkiye; mucahit.karaduman@ozal.edu.tr; 3 Department of Artificial Intelligence and Data Engineering, Firat University, 23119 Elazig, Türkiye; 4 Department of Biostatistics and Medical Informatics, Faculty of Medicine, Inonu University, 44280 Malatya, Türkiye

**Keywords:** alcoholic hepatitis, alcoholic cirrhosis, HBV-related cirrhosis, computed tomography, deep learning, hybrid convolutional neural network, attention mechanism

## Abstract

**Background:** Differentiating alcohol-related liver disease (ARLD) from chronic liver injury caused by other etiologies remains a clinically relevant challenge in cross-sectional imaging. In particular, alcoholic hepatitis and alcoholic cirrhosis may overlap morphologically with HBV-related cirrhosis on CT imaging. To develop and assess an attention-residual hybrid Convolutional Neural Network (CNN) for multiclass CT image classification of alcoholic hepatitis, alcoholic cirrhosis, HBV-related cirrhosis, and living liver donors. **Methods**: A four-class liver image dataset comprising 5760 CT images from 144 individuals (36 per group) was constructed using images from alcoholic hepatitis, alcoholic cirrhosis, HBV-related cirrhosis, and living liver donors. The dataset was partitioned into training, validation, and test sets at the patient level. Five pretrained CNN architectures, including DenseNet121, ResNet50, MobileNetV3-Large, EfficientNetB0, and ConvNeXt-Tiny, were first fine-tuned and comparatively evaluated. Based on F1-score ranking, DenseNet121 and ConvNeXt-Tiny were selected as the two backbone networks for the proposed hybrid model. The final architecture integrated Attention Pooling, Feature-wise Linear Modulation (FiLM), Multi-head Attention, Gated Linear Units, Residual Connections, and layer normalization to improve feature fusion and contextual representation. **Results**: The proposed model demonstrated the best overall performance among all evaluated architectures on the test set. It achieved an accuracy of 99.55%, a weighted F1-score of 99.55%, an MCC of 0.9941, and a Cohen’s kappa coefficient of 0.9940. The model also achieved ROC-AUC and PR-AUC values of 100.00% and 99.99%, respectively, together with an NPV of 99.85%. The proposed model’s performance was also balanced across classes. For Alcoholic Cirrhosis, precision, recall, and F1-score were all 99.64%. For Alcoholic Hepatitis, the corresponding values were 100.00%, 98.93%, and 99.46%, respectively, while HBV-related Cirrhosis achieved 99.29% precision, 99.64% recall, and 99.47% F1-score. Living Liver Donors achieved 99.29% precision, 100.00% recall, and 99.64% F1-score. **Conclusions**: The findings of this exploratory study suggest that routine CT images may contain image-based differences potentially relevant to etiology-oriented classification of diffuse liver disease. Beyond distinguishing ARLD from HBV-related cirrhosis and images from living liver donors, the model also captured image-based differences between major ARLD subgroups, including alcoholic hepatitis and alcoholic cirrhosis. These findings support further investigation of CT-derived image-based differences in larger independent and multicenter datasets.

## 1. Introduction

Chronic liver disease remains a major global health problem and is responsible for more than two million deaths annually, accounting for approximately 4% of all deaths worldwide [1,2]. Among its major etiologies, alcohol-related liver disease (ARLD) contributes substantially to premature mortality and disability; in 2019, ARLD was associated with the loss of 11 million life-years worldwide [3,4]. Contemporary clinical guidelines define ARLD as a broad spectrum ranging from early asymptomatic liver injury to advanced disease with portal hypertension and decompensation [5,6]. Alcoholic hepatitis represents a clinically important inflammatory disease subtype within this spectrum and may present with acute or rapidly worsening jaundice, including severe forms associated with acute-on-chronic liver failure; in severe alcoholic hepatitis, the reported 1-month mortality is approximately 20–50% [5]. Alcoholic cirrhosis, in contrast, reflects chronic structural remodeling and advanced fibrosis. However, these disease subtypes are not strictly mutually exclusive, because alcoholic hepatitis may develop in patients with underlying advanced fibrosis or cirrhosis. In parallel, chronic hepatitis B virus (HBV) infection remains a major global cause of cirrhosis and hepatocellular carcinoma (HCC), affecting approximately 296 million people worldwide and causing an estimated 331,000 deaths from HBV-related cirrhosis in 2019 [7,8,9]. From a clinical perspective, differentiating ARLD from HBV-related cirrhosis remains important, particularly when clinical history is incomplete and imaging findings overlap [10,11].

Assessment of liver disease routinely relies on a combination of biochemical tests and radiologic evaluation to characterize both hepatic function and structural integrity [12,13]. Within this diagnostic framework, cross-sectional imaging modalities such as computed tomography (CT) and magnetic resonance imaging (MRI) play a central role by providing information on parenchymal texture, contour irregularity, nodularity, vascular anatomy, and complications of chronic liver disease [13]. CT is frequently performed in patients with suspected or established liver disease and may reveal morphologic and quantitative features related to steatosis, fibrosis, cirrhosis, and portal hypertension [13]. Nevertheless, conventional visual interpretation has important limitations, and CT may show only moderate sensitivity for compensated cirrhosis [14]. In a prospective cohort study using histology as the reference standard, Hetland et al. [14] reported a sensitivity of 74% and specificity of 93% for CT-based diagnosis of cirrhosis by experienced radiologists, with sensitivity decreasing to 60% in patients with Child–Pugh A cirrhosis. Similarly, Kudo et al. [15] reported an accuracy of 67.0%, sensitivity of 84.3%, and specificity of 52.9% for CT-based diagnostic impression in a multicenter study. Moreover, Cannella et al. [16] demonstrated that the diagnostic performance of conventional morphologic CT assessment may vary according to reader experience and disease etiology, with specificity ranging from 85.7% to 100% across readers and etiologic groups. These findings highlight the limitations and variability of conventional visual CT assessment and provide a clinical reference point for evaluating computational imaging approaches.

Cirrhosis of different etiologies may converge toward similar morphologic patterns, whereas imaging features of alcoholic hepatitis may be subtle, overlapping, or nonspecific outside classic severe presentations [17]. This challenge becomes even more relevant within the ARLD spectrum itself, because alcoholic hepatitis and alcoholic cirrhosis may share imaging characteristics despite representing different clinical and pathological processes [17]. In routine practice, alcohol exposure history may also be incomplete, underestimated, or unreliable, further complicating confident etiologic and clinicoradiologic differentiation [5,18].

More broadly, contemporary phenomics and multimodal phenotyping emphasize the systematic characterization of disease phenotypes by integrating complementary clinical, imaging, molecular, and other biological features rather than relying on a single data source. Such approaches can provide a more comprehensive representation of disease heterogeneity and may facilitate the identification of clinically meaningful subtypes. In liver disease, multimodal and integrative studies combining imaging with clinical and molecular information have similarly sought to refine phenotypic characterization and disease stratification. Within this broader framework, cross-sectional imaging can be regarded as one component of the observable disease phenotype, while quantitative and computational image analysis may enable more detailed characterization of imaging-derived phenotypic differences [19,20,21,22,23].

Beyond routine visual assessment, contemporary liver image analysis includes elastography-based stiffness assessment, radiomics and texture analysis, quantitative morphologic evaluation, and deep learning-based pattern recognition. Depending on the clinical task, these approaches may assess parenchymal attenuation, liver-to-spleen attenuation ratio, surface nodularity, textural heterogeneity, lobar volume redistribution, and imaging correlates of portal hypertension. However, most prior computational studies have focused on fibrosis staging, portal hypertension, or focal lesion characterization rather than multiclass etiologic and clinicoradiologic differentiation within diffuse liver disease. This gap is particularly relevant when the diagnostic task involves overlapping diffuse liver disease patterns rather than a discrete focal lesion [17,24,25,26,27,28].

These diagnostic challenges have stimulated growing interest in computational approaches that can extract clinically relevant information from imaging data beyond routine visual assessment [26,27]. Artificial intelligence-based image analysis, particularly deep learning, has emerged as a promising tool for improving liver imaging diagnosis and differential diagnosis [27,29]. In clinical practice, the challenge is often not simply to recognize an abnormal liver, but to distinguish among disease entities that share overlapping radiologic appearances despite differing in etiology, biological behavior, and clinical implications [13,17]. Convolutional neural networks (CNNs) can automatically learn hierarchical imaging features, but CNNs primarily model local spatial patterns and may be less capable of capturing broader contextual relationships relevant to differential diagnosis [30]. Hybrid architectures that incorporate attention mechanisms and feature-fusion strategies may therefore offer an advantage by integrating complementary information and emphasizing diagnostically relevant regions [30]. However, available evidence in liver imaging remains dominated by fibrosis staging, lesion characterization, and binary classification tasks, whereas multiclass CT-based models specifically designed to differentiate overlapping entities such as alcoholic hepatitis, alcoholic cirrhosis, and HBV-related cirrhosis remain limited [27,29,31].

In this exploratory study, we developed a hybrid deep learning framework to address a clinically meaningful but understudied problem: CT-based multiclass differentiation among alcoholic hepatitis, alcoholic cirrhosis, HBV-related cirrhosis, and living liver donors. Unlike prior studies that have focused mainly on fibrosis staging, lesion characterization, or binary classification, our aim was to investigate whether routine CT images contain discriminative visual patterns that can distinguish ARLD from HBV-related cirrhosis while also differentiating major ARLD subgroups, particularly alcoholic hepatitis and alcoholic cirrhosis. Given the widespread use of CT in routine liver care, such a framework may have potential value as an image-based computer-aided adjunct for liver image interpretation. We further incorporated Grad-CAM as an explainable artificial intelligence tool to visualize image regions contributing to model predictions and to support interpretation of both correctly classified and misclassified cases.

## 2. Materials and Methods

### 2.1. Study Design and Reporting Framework

This study was designed as a retrospective, single-center, image-based classification study conducted at the Inonu University Liver Transplant Institute, Malatya, Türkiye. The source cohort was derived from individuals who underwent liver transplantation or living donor hepatectomy between 1 January 2015 and 31 December 2025. All clinical and imaging data were retrospectively evaluated using the institutional database. The study was structured as an original diagnostic artificial intelligence study focusing on CT image–based classification of ARLD and comparator reference categories.

### 2.2. Study Population and Reference Categories

The final analyzed cohort consisted of 144 individuals, with 36 individuals in each group: living liver donors, individuals with HBV-related cirrhosis, individuals with alcoholic hepatitis, and individuals with alcoholic cirrhosis. Cases were retrospectively selected from the institutional case pool using a random-number-based sampling strategy to reduce selection bias. Eligibility criteria included the availability of non-contrast, arterial, portal venous, and delayed-phase CT images; technically adequate image quality; and a clearly established reference diagnosis. Cases were excluded if any CT phase was missing, if severe motion artifact was present, if the clinical diagnosis was uncertain, if prior liver resection had been performed, or if the available images were insufficient for analysis. After case selection, the final image dataset was organized into four predefined categories and included 5760 images in total: 1440 images from alcoholic hepatitis, 1440 images from living liver donors, 1440 images from HBV-related cirrhosis, and 1440 images from alcoholic cirrhosis.

For retrospective case-definition clarity, the reference categories were operationally defined as follows. The living liver donor group comprised individuals accepted for living donor hepatectomy after standard donor evaluation, with no known chronic liver disease and no clinically significant hepatic abnormality on preoperative assessment [32,33,34,35]. The HBV-related cirrhosis group comprised patients with documented chronic HBV infection and established cirrhosis, based on histopathology when available or on concordant clinical, laboratory, imaging, and, when available, endoscopic findings of cirrhosis and/or portal hypertension [36,37]. The alcoholic hepatitis group comprised patients with documented sustained harmful alcohol use and a clinical-biochemical presentation compatible with widely accepted diagnostic criteria for alcoholic hepatitis, including recent onset or worsening jaundice, serum bilirubin > 3 mg/dL, AST typically 50–400 U/L with an AST/ALT ratio > 1.5, and exclusion of other causes of acute hepatitis [5,38]. The alcoholic cirrhosis group comprised patients with chronic harmful alcohol exposure and established cirrhosis without another dominant etiology, based on histopathology when available or on concordant clinical, laboratory, imaging, and, when available, endoscopic findings. Patients with mixed or uncertain etiologies were not included [5,12,17].

### 2.3. Imaging Source, Acquisition, and Image Extraction

**Figure 1:**
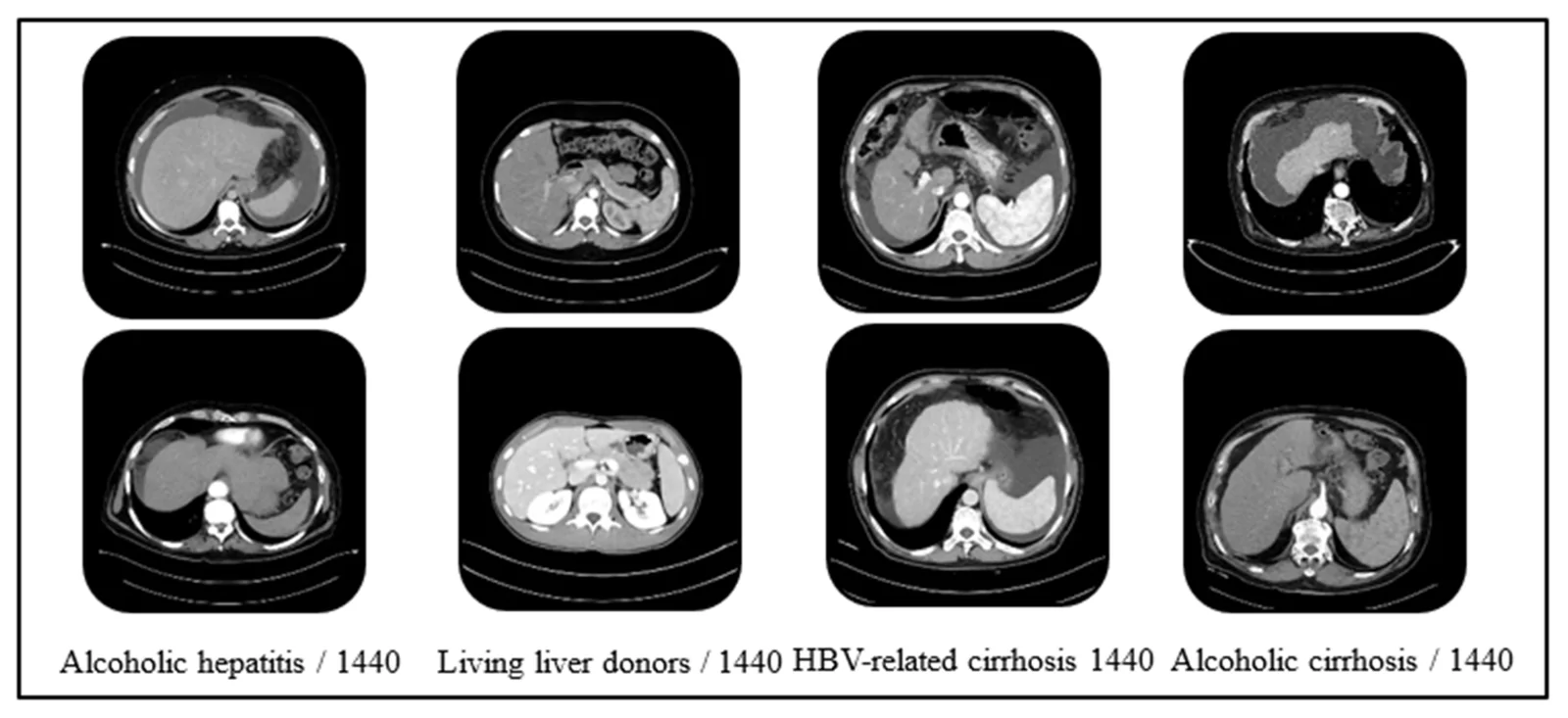
Representative CT images from the four study classes, with the number of images in each class.

This study was based on multidetector computed tomography examinations retrieved from the institutional imaging archive. Each of the 144 individuals was evaluated using non-contrast, arterial, portal venous, and hepatic delayed-phase CT images. CT examinations were obtained using multidetector CT systems, including the Somatom Definition 256-slice scanner or other equivalent clinical-grade systems (Siemens Healthineers, Erlangen, Germany) and the Aquilion 64-slice scanner (Canon Medical Systems, Otawara, Japan). All CT image review, selection, and extraction procedures were performed by the same radiologist using standardized imaging software (SECTRA IDS 7; Sectra AB, Linköping, Sweden) and predefined window settings to ensure consistency and reproducibility. To obtain a balanced image-based dataset, it consists of 1440 CT images for each class. Of these images, 1000 were used for training, 160 for validation, and 280 for testing. The study included 144 patients, with 40 CT images selected from each patient, comprising 10 images from each of the four CT phases. The resulting dataset comprised 5760 CT images. The dataset is divided on a patient-by-patient basis. In total, 100 patients were allocated for training, 16 for validation, and 28 for testing. All CT images were anonymized before model development, and no additional imaging examinations were performed for study purposes. Representative images from each class are shown in Figure 1.

### 2.4. Image Curation and Preprocessing

All CT images were resized according to the input requirements of the selected convolutional neural network architectures, and the final model input size was set to 224 × 224 pixels. Images were prepared in a standardized format before training and testing, ensuring that all baseline and hybrid models were evaluated under the same preprocessing pipeline.

### 2.5. Patient-Level Dataset Partitioning

The dataset comprised data from 144 patients and was divided at the patient level into training, validation, and test subsets comprising 100, 16, and 28 patients, respectively. Patient-level partitioning was stratified by class, with 25, 4, and 7 patients from each class allocated to the training, validation, and test sets, respectively. All images from a given patient were assigned exclusively to a single subset; thus, no patient contributed images to more than one subset. Accordingly, all reported performance metrics reflect classification performance on held-out CT images within the predefined image-based analytical framework.

### 2.6. Baseline CNN Architectures

Five pretrained CNN architectures were evaluated in the initial phase of the study: DenseNet121 [39], ResNet50 [40], MobileNetV3-Large [41], EfficientNetB0 [42], and ConvNeXt-Tiny [43]. These models were selected because they represent widely used and well-established architectures in medical image analysis and provide a suitable benchmark for liver image classification. All models were initialized with ImageNet-pretrained weights and fine-tuned in two stages. In the first stage, only the classification head was trained. In the second stage, all model layers were unfrozen and fine-tuned using the liver image dataset. The performance of these baseline architectures was subsequently compared using the predefined evaluation metrics.

### 2.7. Proposed Hybrid Architecture and Mathematical Formulation

**Figure 2:**
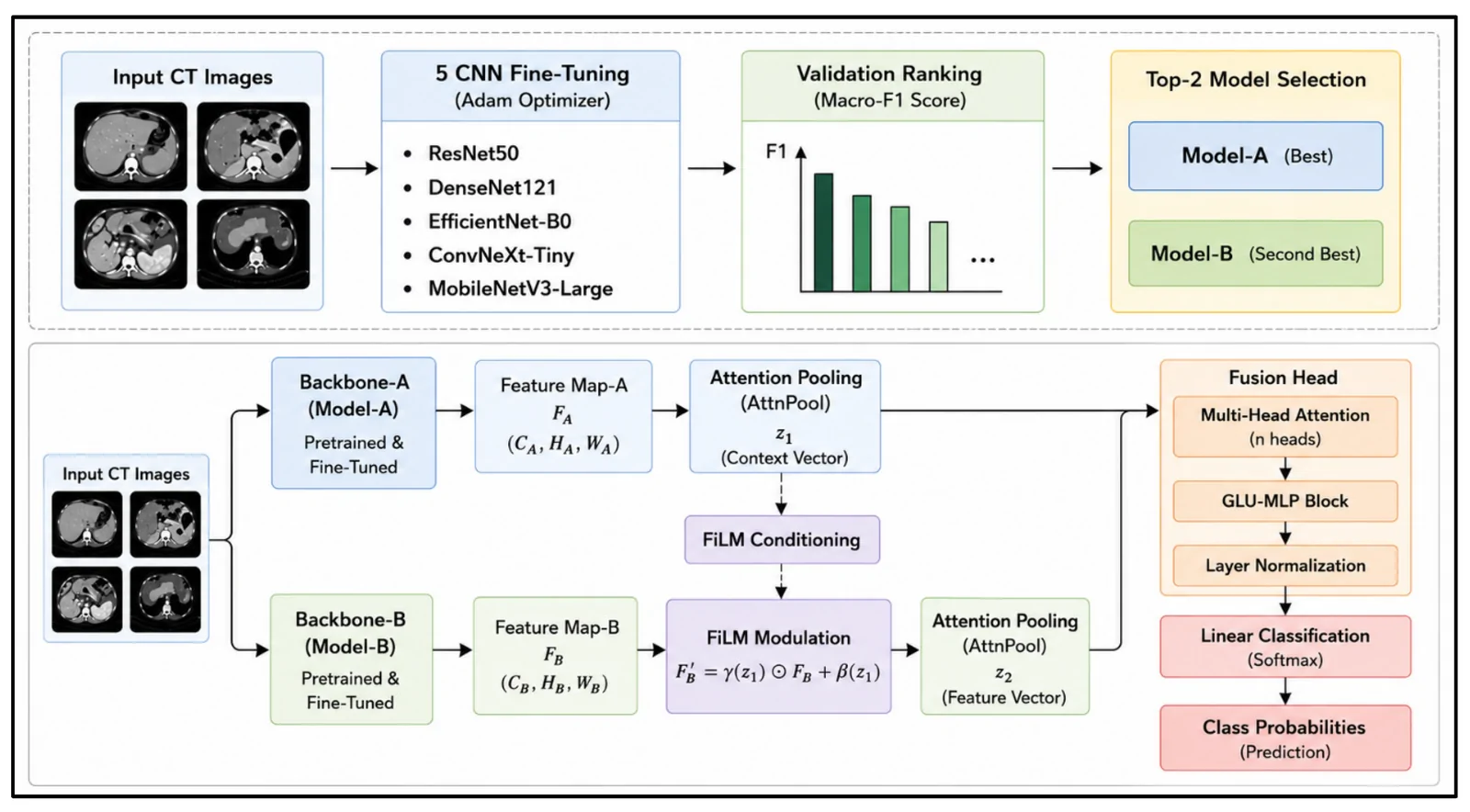
Workflow of the Proposed Attention-Residual Hybrid CNN Model.

The proposed model followed a two-stage development pipeline and its general structure is illustrated in Figure 2. In the first stage, five different CNN architectures were fine-tuned and their classification performance was assessed. Before training, the images were resized according to the input requirements of the corresponding architectures and the dataset was divided into training, validation, and test subsets at the patient level. In the initial step of training, only the classification layer was trained; subsequently, all layers of the model were unfrozen, and the full network was fine-tuned. After all architectures had been evaluated, their F1-scores were compared, and the two best-performing models were selected.

In the second stage, DenseNet121 and ConvNeXt-Tiny, which yielded the highest F1-scores, were used as the backbone networks of the proposed hybrid framework. DenseNet121 was defined as Backbone A and ConvNeXt-Tiny as Backbone B. The input image was simultaneously fed into both backbones for feature extraction. The feature representation obtained from Backbone A was forwarded to the fusion stage after attention pooling. In parallel, the features extracted from Backbone B were conditioned using the features derived from DenseNet121 through Feature-wise Linear Modulation (FiLM) [44]. The FiLM-modulated feature map was then processed by a second attention pooling block. Afterwards, the resulting features were linearly projected, fused, and passed to a multi-head attention (MHA) module. The attention-enhanced representation was subsequently processed by a gated linear unit (GLU)-based multilayer perceptron [45], followed by residual connection, layer normalization, and final softmax classification. Performance evaluation was performed using accuracy, precision, recall, F1-score, and error rate.

To improve the ability of the model to learn deeper contextual relationships, a Transformer-inspired MHA layer was integrated into the hybrid architecture. Although classical convolutional networks are effective in capturing local features, they are more limited in modeling long-range dependencies. Therefore, the attention mechanism was used to re-represent fused features through the query, key, and value matrices.

The mathematical structure of the proposed model can be expressed as follows:

(1) $$F^{A}=B_{A}\left(x\right)\in \;R^{C_{A}xH_{A}xW_{A}}\;$$

where x denotes the input image, $B_{A}$ denotes Backbone A, and $F^{A}$ denotes the extracted feature map with channel number $C_{A}$, height $H_{A}$, and width $W_{A}$.

The extracted feature map was then passed through an attention pooling layer. First, dimensional reduction was performed as

(2) $$U^{A}=\emptyset \left(W_{1}^{A}*F^{A}\right)\;\in \;R^{d_{att}xH_{A}xW_{A}},\;\phi =GELU$$

where $W_{1}^{A}$ denotes the 1 × 1 convolution filter, φ denotes the Gaussian Error Linear Unit activation function, $U^{A}$ denotes the reduced output feature tensor, and $d_{att}$ denotes the attention feature dimension.

Next, the attention logits were computed as

(3) $$S^{A}=W_{1}^{A}*U^{A}\;\in \;R^{1xH_{A}xW_{A}}$$

The normalized attention coefficients were then obtained as

(4) $$\alpha_{ij}^{A}=\frac{exp(S_{ij}^{A})}{\sum_{p,q}exp(S_{pq}^{A})}$$

where $S^{A}$ denotes the spatial importance score, $\alpha_{ij}^{A}$ denotes the normalized attention coefficient at position (i, j), and p and q denote the summation indices over all spatial positions.

The context vector was then generated by weighted aggregation:

(5) $$z_{1}=\sum_{i,j}\alpha_{ij}^{A}U_{:,ij}^{A}\;\;\in \;R^{d_{att}}$$

where $U_{:,ij}^{A}$ denotes the local feature vector at position (i, j) and $z_{1}$ denotes the resulting context vector.

For Backbone B, features were likewise extracted according to Equation (1). After the context vector had been produced from Backbone A, FiLM conditioning was applied to Backbone B in order to perform channel-wise scaling and shifting. The scale and shift vectors were computed as

(6) $$\gamma =W_{\gamma }^{\left(2\right)}\phi \left(W_{\gamma }^{\left(1\right)}z_{1}\right)\;\in \;R^{C_{B}}$$

(7) $$\beta =W_{\beta }^{\left(2\right)}\phi \left(W_{\beta }^{\left(1\right)}z_{1}\right)\;\in \;R^{C_{B}}$$

where γ denotes the channel-wise scale vector, β denotes the channel-wise shift vector, $W_{\gamma }^{\left(1\right)}$ and $W_{\gamma }^{\left(2\right)}$ denote linear layers, and φ again denotes the GELU activation function.

The FiLM modulation itself was defined as

(8) $${\tilde{F}}_{cij}^{B}=\left(1+\gamma_{c}\right)F_{Cij}^{B}+\beta_{c},\;{\tilde{F}}^{B}\in \;R^{C_{B}xH_{B}xW_{B}}$$

where ${\tilde{F}}^{B}$ denotes the FiLM-modulated feature map. After this step, the modulated Backbone B feature map was again passed through the attention pooling layer using the same reduction, attention-logit, soft-attention, and weighted-aggregation operations defined in Equations (2)–(5), thereby producing the corresponding context representation for the second pathway.

After feature fusion, the MHA calculation was defined as

(9) $$Attention\left(Q,K,V\right)=softmax\left(\frac{QK^{T}}{\sqrt{d_{k}}}\;\right)V$$

where Q denotes the query matrix, K denotes the key matrix, V denotes the value matrix, and $d_{k}$ denotes the key dimension. In this formulation, Q is used by the model to query the relationships of a feature position with other positions, K provides the reference representation of each position, and V carries the information content that is weighted according to the attention scores.

The multi-head version was formulated as

(10) $$MHA\left(Q,K,V\right)=\left[{head}_{1};\ldots \ldots .;{head}_{h}\right]W^{O}$$

where h denotes the number of attention heads, ${head}_{i}$ denotes the output of the i-th attention head, and $W^{O}$ denotes the output projection matrix. In this multi-head structure, multiple query-key-value groups are computed in parallel so that different heads can learn different relational patterns and feature subspaces. After the MHA block, the resulting representation was passed through the GLU-MLP layer and then classified by the final softmax layer.

### 2.8. Training Strategy, Hyperparameters, and Implementation Environment

For the baseline CNN models, the classifier head was trained for 5 epochs, followed by 12 epochs of full-network fine-tuning. For the proposed hybrid model, the feature dimension in the FiLM conditioning and attention pooling layers was set to 256, the fusion embedding dimension was 256, and the multi-head attention module used 4 attention heads. The proposed hybrid model was trained for 25 epochs. All models were trained using the Adam optimizer with an initial learning rate of 1 × 10^−4^ and a batch size of 32. The backbone networks were initialized with ImageNet-pretrained weights and fine-tuned using the selected learning rate throughout training. All experiments were conducted in Google Colab using NVIDIA T4 GPU hardware.

### 2.9. Performance Metrics and Statistical Analysis

The classification performance of each model was evaluated using accuracy, balanced accuracy, precision, recall, specificity, macro F1-score, weighted F1-score, and confusion matrices. Additional performance measures included the Matthews Correlation Coefficient (MCC), Cohen’s kappa coefficient, Receiver Operating Characteristic Area Under the Curve (ROC-AUC), Precision–Recall Area Under the Curve (PR-AUC), and Negative Predictive Value (NPV). Class-specific precision, recall, and F1-score values were also calculated for the proposed hybrid model. Predictive probability calibration was assessed using the Expected Calibration Error (ECE), Brier score, and Logarithmic Loss (Log-Loss). Bootstrap resampling was used to calculate 95% confidence intervals for selected performance metrics. To compare the proposed model with the baseline architectures on the same test images, paired classification outcomes were evaluated using the McNemar test. Holm correction was applied to adjust *p*-values for multiple comparisons, and a two-sided adjusted *p*-value < 0.05 was considered statistically significant.

### 2.10. Explainability and Additional Analyses

Gradient-weighted Class Activation Mapping (Grad-CAM) was used as an explainable artificial intelligence tool to identify the image regions that contributed most strongly to the model predictions and to facilitate the interpretation of misclassified cases. Grad-CAM maps were generated for representative correctly classified test images from each class and for selected misclassified test images, and the analysis was used as a qualitative visual aid rather than as a quantitative localization method.

### 2.11. Study Protocol and Ethical Considerations

This study was conducted in accordance with the ethical principles of the Declaration of Helsinki (1964) and its subsequent amendments, and complied with all applicable institutional and national regulations governing research involving human participants. Ethical approval was obtained from the Inonu University Institutional Review Board (IRB) for Non-Interventional Studies (session date: 24 February 2026; session number: 4; decision number: 2026/9464). As the study had a retrospective design involving review of medical records and re-evaluation of archived radiological images, the requirement for individual informed consent was waived. The study was designed, conducted, analyzed, and reported in accordance with the STROBE (Strengthening the Reporting of Observational Studies in Epidemiology) guidelines to enhance transparency, reproducibility, and methodological rigor.

## 3. Results

### 3.1. Cohort and Dataset Composition

The dataset consists of four classes and contains 5760 CT images. In each class, 1000 images were used for training, 160 for validation, and 280 for testing. A total of 1440 images are available in each class. The final cohort analyzed consisted of 144 individuals in total, with 36 individuals in each of the four study categories. Each individual provided contrast-free, arterial, portal venous, and hepatic delayed-phase CT images.

### 3.2. Performance Comparison of Proposed Model and Baseline Models

To compare the performance of the proposed model, results were also obtained with the DenseNet121, ConvNext-Tiny, ResNet50, MobileNetV3-Large, and EfficientNetB0 models. The proposed model is based on the two models that yielded the best results according to the validation results. The validation results obtained from the pre-trained models are presented in Table 1.

Table 1 shows that the most successful model is DenseNet121 with 98.59% validation accuracy. This is followed by ConvNext-Tiny with 97.97%, MobileNetV3-Large with 95.94%, ResNet50 with 95.63%, and EfficientNet-B0 with 94.22%. Therefore, the proposed model, DenseNet121, and ConvNext-Tiny were used as the backbone. The test performance metrics obtained from the proposed and pre-trained models are presented in Table 2.

Table 2 shows that the most successful model is the proposed model with an accuracy of 99.55%. Among the pre-trained models, the DenseNet-121 and ConvNeXt-Tiny models, used as backbones in the proposed model, achieved the strongest accuracy values. The lowest accuracy value among the pre-trained models was obtained in the EfficientNet-B0 architecture with 94.02%. Weighted F1, Matthews Correlation Coefficient (MCC), Cohen’s Kappa ROC-AUC, PR-AUC, and Negative Predictive Value (NPV) metrics were also used in the study to measure the performance of the models. The performance measurement metrics obtained in the pre-trained and proposed models are presented in Table 3.

Table 3 shows that the proposed model achieves more successful results than pre-trained models in all metrics. The proposed model obtained a Weighted F1 score of 99.55%, an MCC of 0.9941, and a Cohen’s Kappa value of 0.9940. Furthermore, the ROC-AUC of 100.00% and PR-AUC of 99.99% demonstrate the high discrimination power of the model between classes. The NPV of 99.85% for the proposed model indicates a very low probability of false negative predictions in negatively classified samples. Among the pre-trained models used in the study, DenseNet121 showed the closest performance to the proposed model with a weighted F1 score of 98.75%, an MCC of 0.9834, and a Cohen’s Kappa value of 0.9833, while EfficientNet-B0 yielded the lowest overall results.

Table 4 presents the Expected Calibration Error (ECE), Brier score, and logarithmic loss (Log-Loss) values to evaluate the predictive reliability and probability calibration of the models. Lower values in all these measures indicate better calibration performance. Table 4 presents the calibration performance values of the proposed model and pre-trained models.

Table 4 presents the ECE, Brier score, and Log-Loss values to evaluate the prediction reliability and probability calibration of the models. Low values for these metrics are important for the performance of the models. Among the models used in the study, the most successful results were obtained in the proposed model. The proposed model achieved an ECE of 2.88%, a Brier score of 0.0084, and a Log-Loss of 0.0397. Specifically, the lower ECE value of the proposed model compared to other models indicates that the prediction probabilities generated by the model show a higher level of agreement with the observed results. Similarly, a low Brier score supports the closeness of probabilistic predictions to the actual class labels, while a low Log-Loss value indicates that the model’s erroneous and high-confidence predictions remain at a lower level. Among the pre-trained models used in the study, DenseNet121 was the most successful in terms of Brier score and Log-Loss, while EfficientNet-B0 showed the highest error values in all three calibration metrics. When the calibration values obtained in Table 4 are evaluated together with previous classification performance results, it is revealed that the proposed model not only provides high classification success but also produces more reliable and well-calibrated prediction probabilities.

In class-based performance evaluation, examining precision, recall, and the F1 score together is crucial for assessing the models’ performance in each class. Precision indicates the extent to which samples assigned to a particular class are correctly classified, recall shows the degree to which the model can identify actual samples belonging to that class, and the F1 score calculates the overall class-level performance by considering the balance between precision and recall. The support value shows the number of samples evaluated for each class, allowing for the interpretation of the performance results within the context of data distribution. Table 5 presents the class-specific performance measurement metrics of the proposed model.

Table 5 shows that the proposed model exhibits high and balanced classification performance in all four classes. In the Alcoholic Cirrhosis class, precision, recall, and F1-score all reached 99.64%. Achieving 100% precision in the Alcoholic Hepatitis class indicates that no false positive predictions were made in the test images assigned to this class, while the 98.93% recall rate suggests that some genuine Alcoholic Hepatitis test images were confused with other classes. In the HBV-related Cirrhosis class, high and balanced performance was achieved with 99.29% precision, 99.64% recall, and 99.47% F1-score. In the Living Liver Donors class, achieving 100% recall shows that all test images belonging to this class were correctly identified. Furthermore, the presence of an equal number of samples (*n* = 280) in each class indicates that the class-based performance results were obtained on a balanced test distribution and that the comparison between classes was not affected by differences in data volume. Overall, F1 scores ranging from 99.46% to 99.64% demonstrate that the proposed model exhibits consistent performance across classes in distinguishing different liver conditions.

The 95% confidence intervals calculated using the bootstrap method are important for evaluating the stability of the model’s performance values against sample variability and the level of uncertainty in the predictions. While a single-point estimate summarizes the model performance, confidence intervals provide additional information about the statistical reliability of the results by showing in which range the actual performance is expected to be found. Narrower confidence intervals, in particular, indicate lower uncertainty in performance predictions and greater stability of model results against sample variations. The bootstrap 95% confidence intervals for the proposed model are presented in Table 6.

Table 6 shows that the proposed model exhibits high point estimates and narrow 95% confidence intervals in its performance metrics. Accuracy was calculated as 99.55%, and the 95% confidence interval was obtained as 99.11–99.91%. Similarly, the Balanced Accuracy and Macro-F1 values of 99.55%, and the lower confidence limits remaining above 99%, indicate that high performance is maintained not only in terms of overall accuracy but also in terms of balanced success between classes. The 100.00–point estimate and the very narrow confidence interval of 99.99–100.00% obtained for ROC-AUC demonstrate the model’s high capacity to distinguish between classes. Overall, the narrow confidence intervals and the high performance level even at the lower limits show that the results obtained by the proposed model are stable against sampling variability.

To assess whether the observed performance differences between the models were statistically significant, the predictions of the proposed model and the reference models on the same test images were compared using the McNemar test. The McNemar test evaluates the difference in error behavior between two classifiers by considering pairs of inconsistent samples where only one of the models correctly classified. Holm correction was applied to the *p*-values to control for the increase in the Type I error rate in comparisons performed with multiple reference models. Thus, the statistical significance of the observed performance superiority of the proposed model compared to the reference models was evaluated by considering multiple comparisons. The statistical comparison between the proposed model and the basic models is presented in Table 7.

Determining whether the observed performance differences between the models are statistically significant is important to ensure that the superiority of the proposed approach is not based solely on numerical performance values. For this purpose, paired classification results obtained on the same test samples were compared using the McNemar test. In addition, Holm correction was applied to control for the Type I error rate that may arise from comparisons with multiple reference models. Thus, the statistical reliability of the performance differences between the proposed model and the reference models was evaluated by considering multiple comparisons. The results of the statistical comparison between the proposed model and the base models are presented in Table 7.

**Figure 3:**
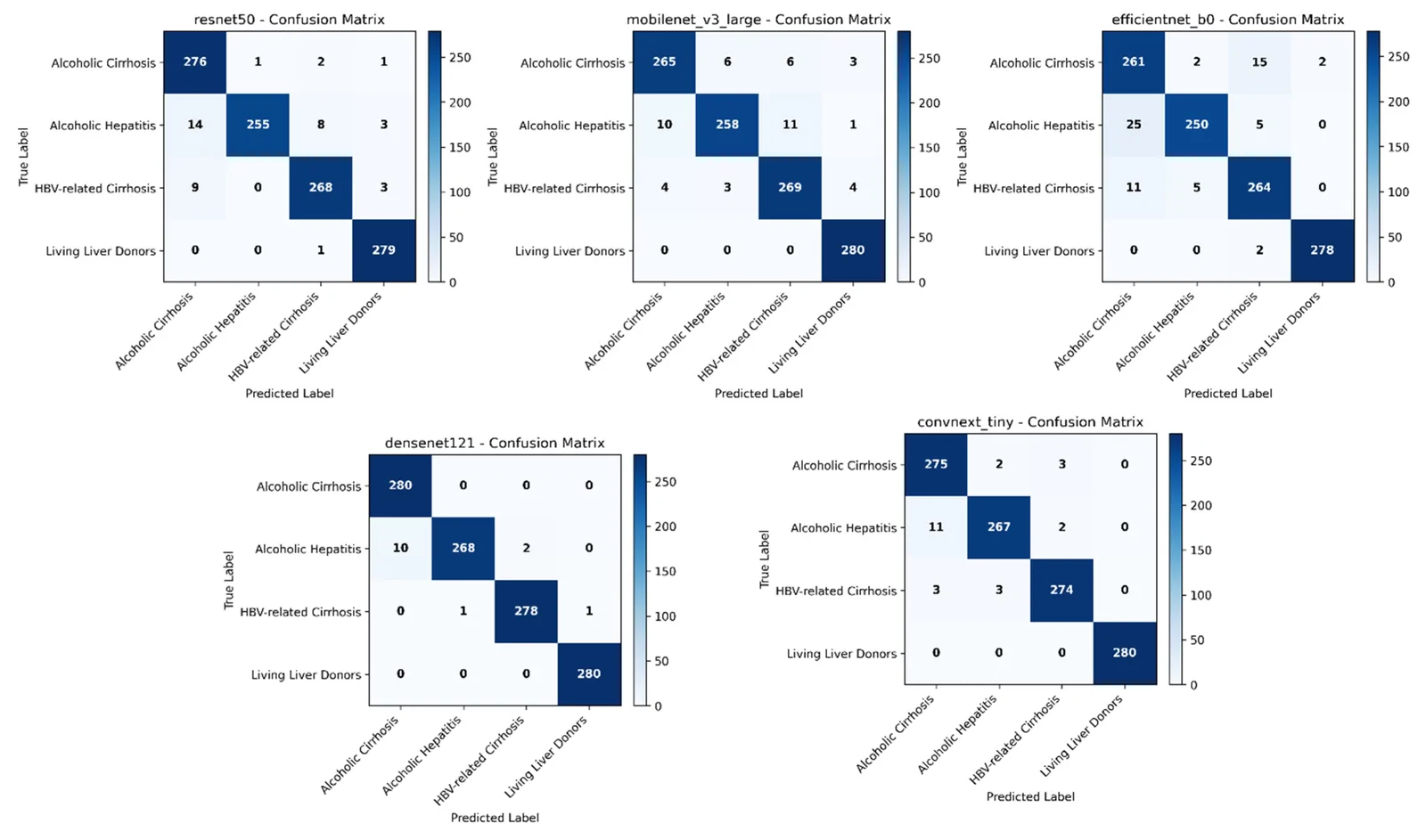
Confusion matrices of the baseline deep learning models.

Table 7 shows that the proposed model achieved statistically significant better results compared to all reference models. The fact that all *p*-values remained below 0.05 after Holm correction indicates that the observed differences retain their significance even when multiple comparisons are considered. To compare the performance of the proposed model, the confusion matrices obtained from the pre-trained models used in the study are presented in Figure 3.

**Figure 4:**
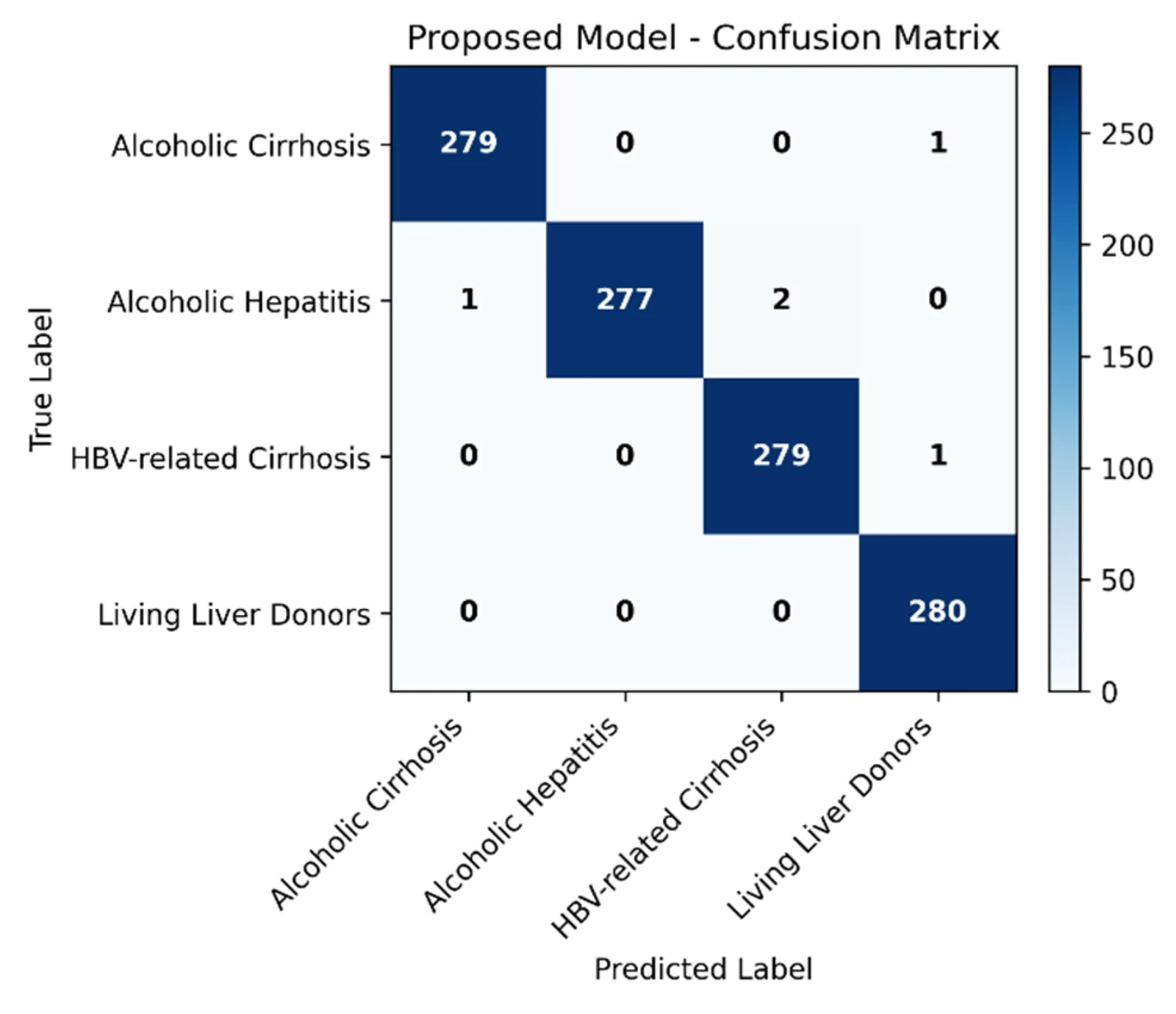
Confusion matrix of the proposed model.

Figure 3 shows that pre-trained models generally demonstrated high classification accuracy in all four classes, but error distributions differed among the models. In the ResNet50, MobileNetV3-Large, and EfficientNet-B0 models, errors were particularly concentrated in the classification of Alcoholic Hepatitis as Alcoholic Cirrhosis. EfficientNet-B0 classified 15 of the Alcoholic Cirrhosis samples as HBV-related Cirrhosis, showing a more significant confusion between these two cirrhosis classes compared to the other models. DenseNet121 and ConvNeXt-Tiny exhibited more balanced classification performance with lower error rates. Specifically, DenseNet121 correctly classified all test images in the Alcoholic Cirrhosis and Living Liver Donors classes, while ConvNeXt-Tiny achieved error-free classification in the Living Liver Donors class. The confusion matrix obtained in the proposed model is presented in Figure 4.

Figure 4 shows that the proposed model correctly predicted 279 out of 280 Alcoholic Cirrhosis test images, while incorrectly predicting 1 image as Living Liver Donors. The proposed model incorrectly predicted 3 test images in the Alcoholic Hepatitis category and 1 test image in the HBV-related category. The proposed model correctly predicted all test images in the Living Liver Donors category. Out of 1120 test images, the proposed model correctly predicted 1115 and incorrectly predicted 5.

### 3.3. Ablation Studies and Robustness Analysis

Ablation analysis is crucial for individually evaluating the contribution of the core components of the proposed architecture to model performance and for justifying the final architectural design. In this context, different architectural structures were compared, using only the core models and incorporating parallel feature combining, multi-head attention, FiLM, and attention-based pooling components. The combined evaluation of Accuracy, Balanced Accuracy, Macro Precision, Macro Recall, Specificity, and Macro-F1 metrics allows for the examination of the impact of each architectural change on overall performance as well as on the success of balanced classification between classes. Ablation studies of the proposed model are presented in Table 8.

According to the ablation results presented in Table 8, the proposed model, including all components, achieved the highest overall performance with 99.55% Accuracy, 99.55% Balanced Accuracy, 99.56% Macro Precision, 99.85% Specificity, and 99.55% Macro-F1 values. When the spine models were evaluated separately, DenseNet121 reached 98.75% and ConvNeXt-Tiny reached 97.86% Macro-F1 values. In contrast, combining the features obtained from the two architectures in parallel increased the Macro-F1 value to 99.37%, demonstrating that the combined use of complementary features contributed to the classification performance. Macro-F1 values of 99.20%, 99.01%, and 98.84% were obtained in the Multi-Head Attention, FiLM, and Attention Pooling variants, respectively. The fact that the final model outperforms all of these variants supports the idea that the combined use of components in the proposed architecture provides a more effective feature representation.

Robustness analysis is important for evaluating a model’s performance under ideal test conditions, as well as its ability to maintain its performance in the face of changes in image quality and imaging conditions. Since images in real-world applications can be subject to noise, blurring, brightness and contrast changes, or compression-induced distortions, evaluations performed under such controlled perturbations provide additional information about the model’s stability and generalizability. For this purpose, the performance of the proposed model was evaluated under Gaussian noise, Gaussian blurring, brightness and contrast changes, and JPEG compression using Accuracy, Balanced Accuracy, Macro-F1, ROC-AUC, and PR-AUC metrics. The obtained metrics are presented in Table 9.

Table 9 reveals that the proposed model exhibits varying levels of stability against different image distortions. The proposed model achieved 99.55% Accuracy and Macro-F1 values in the original images, while maintaining 99.46% accuracy with a brightness change of 0.75, 99.38% with a contrast change, and 98.93% under JPEG compression. These results demonstrate the high stability of the proposed model against brightness and contrast changes and moderate JPEG compression. When Gaussian noise is added, although Macro-F1 decreases to 90.91%, the ROC-AUC remains at 99.67% and PR-AUC at 98.77%, preserving the model’s discrimination capacity. The most significant performance loss occurred under Gaussian blurring. Accuracy dropped to 67.95% and Macro-F1 to 65.04%, while ROC-AUC was obtained at 95.34% and PR-AUC at 87.77%. This finding indicates that the proposed model is particularly sensitive to spatial detail loss caused by blurring. Overall, the results show that the model exhibits high robustness against photometric changes and image compression, but noise and especially blurring-related degradations have a more significant impact on classification performance.

### 3.4. Grad-CAM Findings

**Figure 5:**
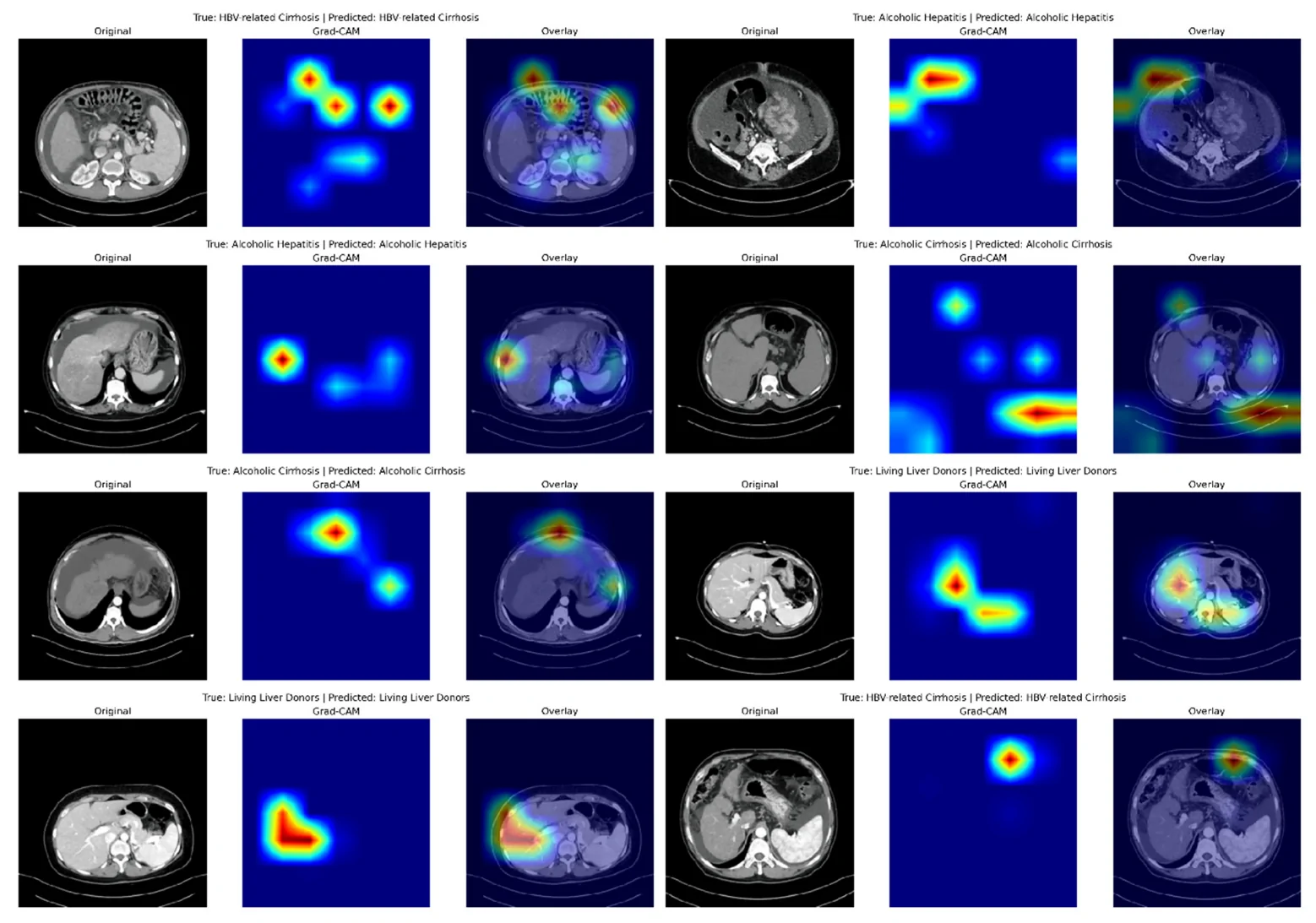
Grad-CAM visualizations of the proposed model for the four classes.

Despite the high classification performance of deep learning models, the inability to directly interpret their decision-making mechanisms constitutes a significant limitation, particularly in medical imaging applications. Therefore, the Gradient-weighted Class Activation Mapping (Grad-CAM) method was used to visually examine the image regions contributing to the classification decisions of the proposed model. Grad-CAM projects model activations related to the target class onto the image, allowing for the identification of spatial regions that are more influential in the prediction process. Figure 5 shows the Grad-CAM results for correctly classified samples belonging to the Alcoholic Cirrhosis, Alcoholic Hepatitis, HBV-related Cirrhosis, and Living Liver Donors classes.

**Figure 6:**
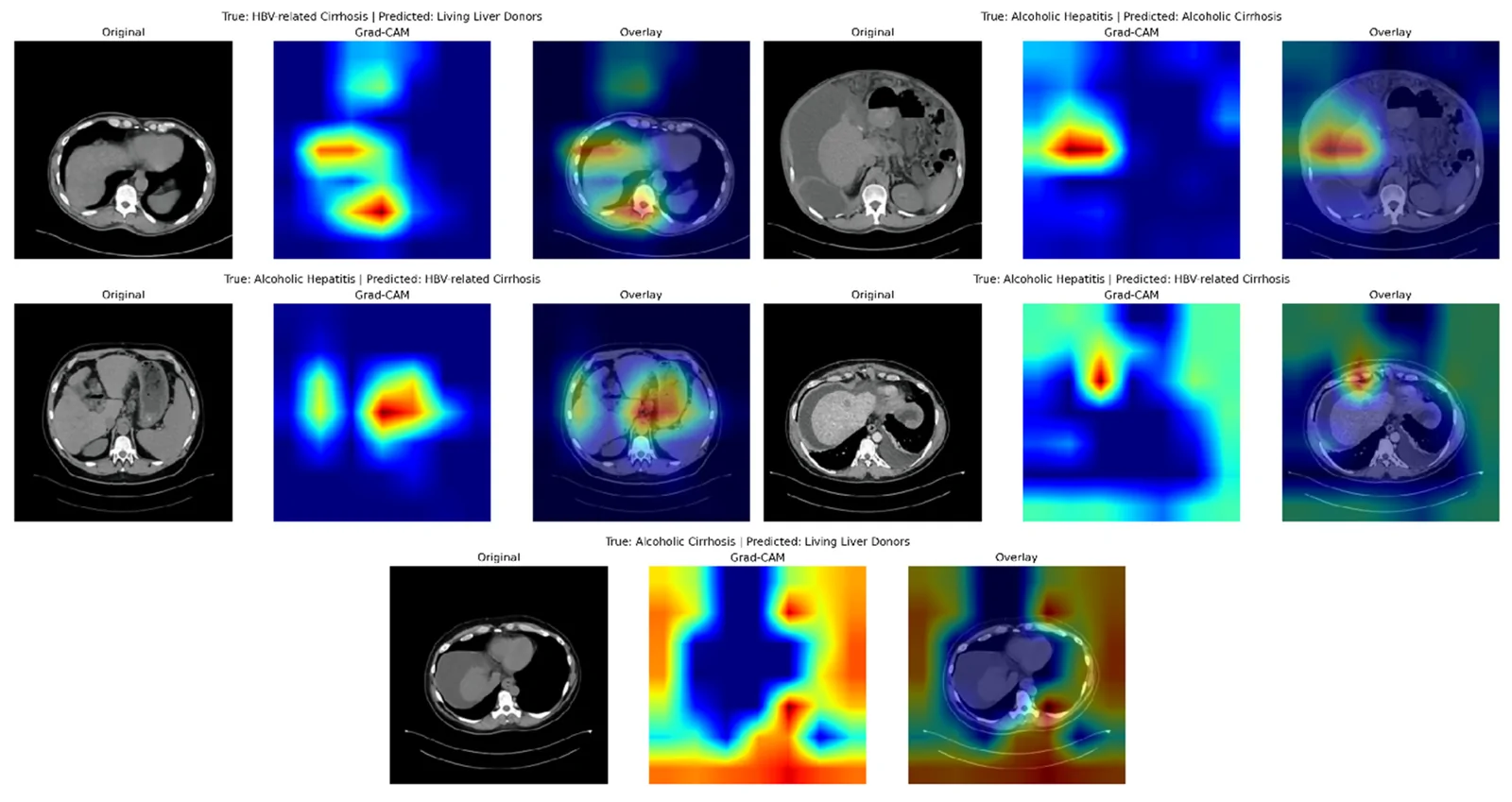
Grad-CAM visualizations of misclassified samples by the proposed model.

Figure 5 shows the Grad-CAM results for correctly classified samples belonging to the Alcoholic Cirrhosis, Alcoholic Hepatitis, HBV-related Cirrhosis, and Living Liver Donors classes. The visualizations show that the model utilizes spatial features from different anatomical regions in its decision-making process, and that activations are concentrated in the liver and surrounding abdominal regions in many samples. However, activation distributions vary between samples, and in some images, regions outside the liver also contribute to the model’s decision. This suggests that the model’s classification decisions are not based on a single local region and that it can utilize different spatial features. When considered together with the fact that all samples from the four classes were correctly classified, the Grad-CAM results provide complementary explanatory capabilities for visually examining the prediction mechanism of the proposed model and revealing the regions that influence classification decisions. Grad-CAM visualizations of the samples misclassified by the proposed model are presented in Figure 6.

Figure 6 presents Grad-CAM examples of samples misclassified by the proposed model. Erroneous predictions were observed among the Alcoholic Cirrhosis, Alcoholic Hepatitis, HBV-related Cirrhosis, and Living Liver Donors classes. While activations were concentrated in and around the liver in some samples, they were spread across larger abdominal areas or regions outside the liver in others. The absence of a distinct and localized activation pattern in some misclassified images suggests that the model may have made erroneous decisions when it failed to adequately represent the distinguishing features. However, since Grad-CAM visualizations alone do not provide a causal explanation, these activation patterns should be considered as visual indicators of the model’s decision-making mechanism. This error analysis shows that despite the generally high classification performance of the proposed model, similar image features between classes and features obtained from regions outside the liver may have contributed to erroneous predictions in some samples.

## 4. Discussion

In this proof-of-concept study, the findings suggest that routine CT images may contain image-based differences potentially relevant to multiclass differentiation of diffuse liver disease. These differences may extend beyond the distinction between ARLD and HBV-related cirrhosis and may also be present between major ARLD subgroups, including alcoholic hepatitis and alcoholic cirrhosis. Within this image-based framework, the proposed attention-residual hybrid CNN achieved the strongest overall performance among all evaluated architectures, with 1115 of 1120 test images correctly classified. Taken together, these findings support the concept that routine CT images may contain discriminative visual information relevant to etiology-oriented classification of diffuse parenchymal liver disease.

This clinical framing also clarifies where the present study sits within the broader liver imaging artificial intelligence literature. Most AI studies in hepatic imaging have focused on focal liver lesions, especially lesion detection, primary liver tumors, radiomics-based tumor characterization, and treatment-response assessment. Even within diffuse liver disease, the literature has been weighted more heavily toward fibrosis staging, cirrhosis detection, steatosis quantification, and portal hypertension assessment than toward etiologic differential diagnosis across overlapping chronic liver disorders [25,26,27,29]. Thus, the key gap is not the absence of liver imaging AI altogether, but the relative scarcity of studies addressing etiology-oriented classification in diffuse parenchymal liver disease.

Within CT specifically, the literature has been dominated by studies asking whether hepatic steatosis, liver fibrosis, or cirrhosis is present, rather than whether one diffuse liver disease etiology can be distinguished from another. For steatosis, Pickhardt et al. [46] reported an AUC of 0.938 for detecting at least moderate hepatic steatosis using an automated artificial intelligence attenuation tool on portal venous phase contrast-enhanced CT, while Kim et al. [47] showed that deep learning-based automated attenuation measurements achieved an AUC of 0.868, with the liver-spleen attenuation difference outperforming liver attenuation alone. For fibrosis, Choi et al. [48] reported AUROCs of 0.96, 0.97, and 0.95 using a CT-based deep learning system, whereas Yasaka et al. [49] used a deep convolutional neural network and reported more modest AUC values of 0.74, 0.76, and 0.73 for the same thresholds. For cirrhosis, Pickhardt et al. [50] showed that semiautomated liver surface nodularity quantification on MDCT achieved an AUC of 0.959 for cirrhosis, while Mathai et al. [51] reported an AUC of 0.877 using a fully automated and explainable CT-based liver surface nodularity technique.

A smaller group of studies has come closer to etiology-oriented classification in liver disease. Elkilany et al. [52] showed on gadoxetic acid-enhanced MRI radiomics that cirrhosis etiology could be differentiated across multiple causes, with the strongest performance for cholestatic versus noncholestatic cirrhosis (AUC 0.960 for 2D models and 0.910 for 3D models; accuracy 87.6% and 85.6%, respectively). Luetkens et al. [53] demonstrated that deep learning on standard MRI could distinguish alcoholic from other-than-alcoholic cirrhosis, with an AUC of 0.82 and an accuracy of 0.75. In a CT-based ARLD study, Tana et al. [54] reported that a texture-based model differentiated acute alcohol-associated hepatitis from controls with an accuracy of 82.4%, whereas a two-dimensional dense CNN achieved 70.0%. Taken together, these studies support the broader premise that diffuse liver diseases can carry etiology-related imaging signatures. At the same time, they largely reflect MRI-based approaches, binary classification designs, or comparisons between acute alcohol-associated hepatitis and controls, leaving a more specific gap for CT-based multiclass differentiation among alcoholic hepatitis, alcoholic cirrhosis, and HBV-related cirrhosis. In this context, the present study extends the literature by addressing this narrower but clinically relevant differential diagnosis problem. More specifically, compared with the MRI-based binary classification approach of Luetkens et al. [53], the present study uses CT and expands the task to a four-class framework comprising alcoholic hepatitis, alcoholic cirrhosis, HBV-related cirrhosis, and living liver donors. In addition, rather than relying solely on conventional pretrained CNN classifiers, the present study evaluates a hybrid attention-residual architecture integrating complementary feature representations. Direct numerical comparison across these reports should nevertheless be made cautiously, because the underlying tasks, disease definitions, imaging modalities, study populations, and evaluation units were not identical.

When interpreted within this problem setting, the favorable image-level performance of the proposed model may plausibly relate in part to its hybrid design. DenseNet121 and ConvNeXt-Tiny were selected as complementary backbone networks after comparative evaluation of five pretrained CNN architectures. DenseNet121 promotes feature reuse through dense connectivity, whereas ConvNeXt-Tiny offers a modern convolutional representation with a different feature-extraction profile. The subsequent integration of attention pooling, feature-wise linear modulation, multi-head attention, gated linear units, and residual fusion was intended to preserve local texture information while improving contextual integration across the fused feature space [30]. Conceptually, such a hybrid design is plausible for diffuse liver disease classification, in which diagnostically useful information may arise not only from local parenchymal texture but also from broader contextual relationships and spatial heterogeneity [30,55]. Ablation analysis further showed that the progressive integration of the two backbone networks and additional architectural components was associated with incremental improvements in classification performance, with the complete hybrid architecture achieving the highest overall performance.

The observed misclassifications were limited in number and occurred among the diseased liver categories. Such errors may partly reflect overlapping imaging characteristics between alcoholic hepatitis, alcoholic cirrhosis, and HBV-related cirrhosis. In particular, alcoholic hepatitis and alcoholic cirrhosis are not biologically or morphologically independent entities, because alcoholic hepatitis may occur in patients with underlying advanced fibrosis or cirrhosis, and both conditions may share heterogeneous parenchymal changes related to inflammation, steatosis, fibrosis, and altered liver morphology. Conversely, the complete separation of images from living liver donors from the diseased groups is not unexpected, because CT images from living liver donors lack the structural alterations typically associated with advanced chronic liver injury. Nevertheless, the very small number of misclassified images should be interpreted cautiously, and confirmation in larger and more heterogeneous cohorts remains necessary.

To further explore how the model arrived at its predictions, Grad-CAM was used as an explainable artificial intelligence tool to provide a qualitative interpretation of model behavior by visualizing the image regions that contributed most strongly to each prediction. By generating class-specific heatmaps from the final convolutional feature maps, Grad-CAM helps determine whether the model is focusing on anatomically relevant regions and facilitates interpretation of classification errors [56,57]. In the present study, Grad-CAM was used both to identify the dominant regions of attention in correctly classified images and to aid interpretation of misclassified cases. In correctly classified examples, the model primarily focused on the liver and surrounding upper abdominal anatomical regions, suggesting that the predictions were driven largely by clinically relevant image areas. In some misclassified images, activation patterns were broader and less concentrated over the liver parenchyma, with attention extending to larger abdominal areas or regions outside the liver, which may have contributed to classification uncertainty. These findings support the value of Grad-CAM as an interpretive aid for understanding model attention and error patterns. However, Grad-CAM does not establish that the model has learned clinically causal or pathology-specific features, and the biological significance of these activation maps still requires expert radiologic assessment and more formal localization analyses.

Even with these encouraging findings, the overall interpretation of the present results should remain cautious. Anteby et al. [31], in a systematic review of 16 studies comprising 40,405 radiological images from 15,853 patients, reported that 14 of the 16 studies had substantial concerns regarding risk of bias and applicability. Similarly, Kutaiba et al. [58] identified only six eligible CT-based deep learning studies and found that the reported AUC values ranged widely from 0.63 to 0.97, highlighting both the promise of the field and the magnitude of methodological heterogeneity. These concerns are directly relevant here. Accordingly, the present findings should be regarded as an exploratory demonstration of image-level technical feasibility rather than evidence of ready-to-implement clinical performance. Future work should prioritize validation in larger and independent cohorts, including multicenter datasets, broader clinical heterogeneity, and evaluation strategies designed explicitly to test generalizability beyond the current image-level framework. In addition, multimodal approaches integrating CT findings with demographic, biochemical, histopathological, and clinical severity data may improve both diagnostic robustness and clinical relevance.

### Limitations

This study has several limitations. First, it was a retrospective, single-center analysis conducted in a selected transplant and living-donor population; therefore, spectrum bias may limit generalizability to broader clinical settings. Second, although the dataset was partitioned at the patient level, model predictions and performance metrics were evaluated at the image level, with multiple CT images obtained from each individual. Thus, while no patient contributed images to more than one dataset subset, within-patient correlation among images should be considered when interpreting the image-level performance estimates. Future studies should additionally evaluate patient-level performance using predefined aggregation strategies and statistical approaches that account for within-patient clustering. Third, the present findings were derived from a single institutional dataset and were not externally validated; therefore, the robustness and generalizability of the observed CT-derived visual patterns remain to be confirmed in larger, independent, and multicenter datasets. Fourth, the study did not include comparisons with radiologist interpretation, conventional imaging scores, elastography, serum-based fibrosis indices, or combined clinical models; therefore, the incremental value of the proposed model beyond existing approaches remains uncertain.

## 5. Conclusions

This exploratory image-based study suggests that routine CT may contain discriminative visual information relevant to etiology-oriented classification of diffuse parenchymal liver disease. The proposed attention-residual hybrid CNN identified CT-derived features that distinguished ARLD from HBV-related cirrhosis and living liver donors, while also differentiating major ARLD subgroups, including alcoholic hepatitis and alcoholic cirrhosis. These findings support the presence of potentially distinct CT-based patterns across clinically relevant ARLD subgroups. Larger independent and multicenter datasets, with patient-level evaluation, are needed to assess the robustness, reproducibility, and clinical relevance of these CT-derived signals.

## Figures and Tables

**Table 1:** Validation performance of the candidate backbone models.

Model	Validation Macro-F1 (%)	Validation Accuracy (%)
DenseNet121	98.59	98.59
ConvNeXt-Tiny	97.96	97.97
MobileNetV3-Large	95.92	95.94
ResNet50	95.60	95.63
EfficientNet-B0	94.23	94.22

**Table 2:** Classification performance comparison of the proposed model and baseline networks.

Model	ACC (%)	Balanced ACC (%)	Precision (%)	Recall (%)	Specificity (%)	Macro-F1 (%)	Inference Time (ms/Image)
Proposed Model	99.55	99.55	99.56	99.55	99.85	99.55	22.57
DenseNet121	98.75	98.75	98.78	98.75	99.58	98.75	15.45
ConvNeXt-Tiny	97.86	97.86	97.88	97.86	99.29	97.86	7.01
ResNet50	96.25	96.25	96.38	96.25	98.75	96.24	6.29
MobileNetV3-Large	95.71	95.71	95.72	95.71	98.57	95.70	7.23
EfficientNet-B0	94.02	94.02	94.19	94.02	98.01	94.04	12.82

**Table 3:** Advanced performance comparison of the proposed model and baseline networks.

Model	Weighted F1 (%)	MCC	Cohen’s Kappa	ROC-AUC (%)	PR-AUC (%)	NPV (%)
Proposed Model	99.55	0.9941	0.9940	100.00	99.99	99.85
DenseNet121	98.75	0.9834	0.9833	99.97	99.91	99.59
ConvNeXt-Tiny	97.86	0.9715	0.9714	99.89	99.72	99.29
ResNet50	96.24	0.9505	0.9500	99.72	99.28	98.77
MobileNetV3-Large	95.70	0.9430	0.9429	99.66	99.11	98.58
EfficientNet-B0	94.04	0.9207	0.9202	99.37	98.41	98.01

**Table 4:** Calibration performance of the proposed model and baseline models.

Model	ECE (%)	Brier Score	Log-Loss
Proposed Model	2.88	0.0084	0.0397
DenseNet121	6.29	0.0341	0.0993
ConvNeXt-Tiny	5.38	0.0415	0.1096
ResNet50	6.98	0.0731	0.1638
MobileNetV3-Large	7.08	0.0791	0.1765
EfficientNet-B0	9.15	0.1104	0.2366

**Table 5:** Class-specific performance of proposed model.

Class	Precision (%)	Recall (%)	F1-Score (%)	Support
Alcoholic Cirrhosis	99.64	99.64	99.64	280
Alcoholic Hepatitis	100.00	98.93	99.46	280
HBV-related Cirrhosis	99.29	99.64	99.47	280
Living Liver Donors	99.29	100.00	99.64	280

**Table 6:** Bootstrap 95% confidence intervals of proposed model.

Metric	Estimate	95% CI
Accuracy (%)	99.55	99.11–99.91
Balanced Accuracy (%)	99.55	99.10–99.91
Macro-F1 (%)	99.55	99.10–99.91
Weighted F1 (%)	99.55	99.11–99.91
MCC	0.9941	0.9881–0.9988
ROC-AUC (%)	100.00	99.99–100.00

**Table 7:** Statistical comparison between proposed model and baseline models.

Baseline	Proposed Correct/Baseline Wrong	Proposed Wrong/Baseline Correct	McNemar *p*-Value	Holm-Adjusted *p*-Value
ResNet50	39	2	7.84 × 10^−10^	2.35 × 10^−9^
DenseNet121	11	2	0.0225	0.0225
EfficientNet-B0	63	1	7.05 × 10^−18^	3.52 × 10^−17^
ConvNeXt-Tiny	19	0	3.81 × 10^−6^	7.63 × 10^−6^
MobileNetV3-Large	43	0	2.27 × 10^−13^	9.09 × 10^−13^

**Table 8:** Ablation study of the proposed model.

Variant	ACC (%)	Balanced ACC (%)	Macro Precision (%)	Macro Recall (%)	Specificity (%)	Macro-F1 (%)
Proposed Model	99.55	99.55	99.56	99.55	99.85	99.55
Backbone A only (DenseNet121)	98.75	98.75	98.78	98.75	99.58	98.75
Backbone B only (ConvNeXt-Tiny)	97.86	97.86	97.88	97.86	99.29	97.86
Parallel A + B Fusion	99.38	99.38	99.38	99.38	99.79	99.37
Multi-Head Attention	99.20	99.20	99.20	99.20	99.73	99.20
FiLM	99.02	99.02	99.03	99.02	99.67	99.01
Attention Pooling	98.84	98.84	98.85	98.84	99.61	98.84

**Table 9:** Robustness analysis of proposed model.

Test Condition	ACC (%)	Balanced ACC (%)	Macro-F1 (%)	ROC-AUC (%)	PR-AUC (%)
Original	99.55	99.55	99.55	100.00	99.99
Gaussian Noise (σ = 0.05)	91.16	91.16	90.91	99.67	98.77
Gaussian Blur	67.95	67.95	65.04	95.34	87.77
Brightness (0.75)	99.46	99.46	99.46	100.00	99.99
Contrast (0.75)	99.38	99.38	99.37	100.00	99.99
JPEG Compression (Q = 50)	98.93	98.93	98.93	99.99	99.96

## Data Availability

The data presented in this study are available on request from the corresponding author. The data are not publicly available due to privacy, institutional, and ethical restrictions related to human clinical imaging data.
